# Identification of 2C-B in Hair by UHPLC-HRMS/MS. A Real Forensic Case

**DOI:** 10.3390/toxics9070170

**Published:** 2021-07-15

**Authors:** José Manuel Matey, Adrián López-Fernández, Carmen García-Ruiz, Gemma Montalvo, Félix Zapata, María A. Martínez

**Affiliations:** 1 Department of Chemistry and Drugs, National Institute of Toxicology and Forensic Sciences, Calle José Echegaray 4, Las Rozas de Madrid, 28232 Madrid, Spain; mariaantonia.martinez@justicia.es; 2 Instituto Universitario de Investigación en Ciencias Policiales (IUICP), Universidad de Alcalá, Calle Libreros 27, Alcalá de Henares, 28801 Madrid, Spain; carmen.gruiz@uah.es (C.G.-R.); gemma.montalvo@uah.es (G.M.); 3 Departamento de Química Analítica, Universidad de Alcalá, Química Física e Ingeniería Química Ctra, Madrid–Barcelona km 33,600, Alcalá de Henares, 28871 Madrid, Spain; adrian.lopezf@uah.es; 4 Department of Analytical Chemistry, Faculty of Chemistry, Universidad de Murcia, 30100 Murcia, Spain; felix.zapata@um.es

**Keywords:** 2C-B, hair, “pink cocaine”, UHPLC-HRMS/MS, Orbitrap, hallucinogens, Nexus, NPS

## Abstract

The analysis of drugs of abuse in hair and other biological matrices of forensic interest requires great selectivity and sensitivity. This has been traditionally achieved through target analysis, using one or more analytical methods that include different preanalytical stages, and more complex procedures followed by toxicological laboratories. There is no exception with 2C-series drugs, such as 2C-B, a new psychoactive substance (NPS), which use has emerged and significantly increased, year by year, in the last decades. Continuously new analytical methods are required to selectively detect and identify these new marketed substances at very low concentrations. In this case report, one former case of a polydrug consumer (charged of a crime against public health in Spain) was reanalyzed in hair matrix. In this reanalysis, 2C-B has been positively detected and identified using liquid chromatography coupled to high-resolution mass spectrometry (UHPLC-HRMS/MS). The most selective analytical UHPLC-HRMS/MS method alongside a universal and simpler pretreatment methodology has opened up more possibilities for the detection of substances of different chemical structure and optimization of different HRMS/MS detection approaches allowing the identification of 2-CB in the hair of a real forensic case.

## 1. Introduction

2C-B (4-Bromo-2,5-dimethoxyphenethylamine), also popularly known as Nexus, Venus, Erox, Synergy, Performax, Toonies, or most known as “pink cocaine” (although it is not structurally related to cocaine), is chemically classified as a 2C-type phenethylamine drug with psychedelic and entactogenic properties [1]. It was first synthesized in the 1970s for therapeutic purposes, but then abandoned due to the significant gastrointestinal adverse effects. Compared to other more common psychedelic drugs such as LSD, psilocybin, or 3,4-methylendioxy-N-methyl-amphetamine (MDMA), users described the effects of 2C-B, in a recent controlled study, as non-threatening nor confusing, that the mind keeps clear during the visions and there is no hangover thereafter [2]. Many 2C substances from 2C-Series, such as 2C-B, are listed as Schedule I substances and are considered New Psychoactive Substances (NPS) [3].

Such substances of the 2C series are phenylethylamine-type drugs [4] and can even be subclassified as dimethoxy-phenylethylamines where they share the following core structure proposed by Zapata et al. [5], and are referenced in an article from Maurer’s 2C series [6] (see Table 1).

Among all of them, 2C-B substance is the analogue with the highest prevalence in Spain, as evidenced by the casework at the INTCF, popularly known as “pink cocaine”, “pink powder”, or even “tucibí”.

The European Monitoring Center for Drugs and Drugs Addiction (EMCDDA) Drug Report in 2020 reveals that, from 2008 to 2018, the number of cases involving phenethylamine-derivatives increased almost 5 times [4]. As recently proved by different epidemiological studies [7,8] carried out in some countries, such as the United States and the Netherlands, the consumption of 2C-B has significantly increased over the last decade. The number of cases where 2C-B was seized for identification analysis increased as well. 

From a pharmacodynamics point of view, 2C-B exposure increases neurotransmitters levels in the brain, primarily dopamine and likely also serotonin and norepinephrine, which result in amphetamine-like stimulating effects as well as mescaline-like hallucinogenic effects. All of these (desired and not desired) effects depend on the dose administered [9].

Tiny amounts of the substance are enough to cause psychoactive effects (<10 mg). Therefore, it is sometimes difficult to detect these small quantities on hair with analytical techniques that do not present very low limits of detection and enough selectivity for the mother drugs, and much less for their metabolites. Therefore, this is one of the main reasons why testing these analytes with high-resolution techniques is essential.

So far, there are several studies carried out in hair using high-resolution analytical techniques, such as high-resolution liquid chromatography coupled to mass spectrometry (UHPLC-HRMS/MS) [10,11]. The study of analytical procedures to determine the presence or absence of 2C-B in biological matrices began in the 1990s in rats. The first studies (1998) required the mixed use of both spectroscopic (NMR) and diode array detection (HPLC-DAD) techniques [12]. In 2010, M.R. Meyer and H.H. Maurer studied the metabolism of abuse drugs, with special focus on NPS and compounds of the 2C-X series in human urine, using gas chromatography/mass spectrometry after derivatization. They obtained good results [13]. Later, in 2017, Daniel Pasin et al. tested hallucinogenic phenylethylamines (2C-B and others) by high-resolution mass spectrometry by analysis with non-targeted screening purposes [14]. Different compounds such as ketamine and N,N-dimethyltryptamine tend to have higher levels in hair after chronic consumption. This could be explained due to their relatively high liposolubility (therefore their ability to flow through the keratinocyte cell membrane is higher) and basic pKa, which increases the ionized fraction of compound inside the acidic keratinocyte cytosol, which is not be able to return to the blood plasma [15]. Other parameters seem to affect the concentration in hair, such as the presence of a nitrogen atom in the molecule (which binds with the melanin in hair), and the absence of an acid group, which has been observed to increase the incorporation [16]. Kronstrand et al. also proved that this incorporation is also favoured when the molecule presents N-alkyl chain groups and N-benzene rings [15]. These properties may be of more relevance than half-life and concentrations in plasma. For example, in the study carried out by Nakahara et al. cocaine and benzoylecgonine have lower incorporation in hair despite presenting a higher half-life and plasma concentration than cocaine [17]. In general, this incorporation will also depend on the amounts of consumption for each substance, especially in the cases in which minimum amounts of substance are required, for example, hallucinogens (e.g., LSD).

In the case of 2C-B, there is no information available since no studies with a positive result have been carried out with this drug in hair, at least in the literature revised. In this study, this work will show the pioneer identification of 2C-B in a real hair case, after reanalysis of the hair sample of a known drug user, by UHPLC-HRMS/MS, emphasizing its usefulness for detecting very low concentrations of 2C-B in a real hair sample. 

## 2. Case History 

**Figure 1:**
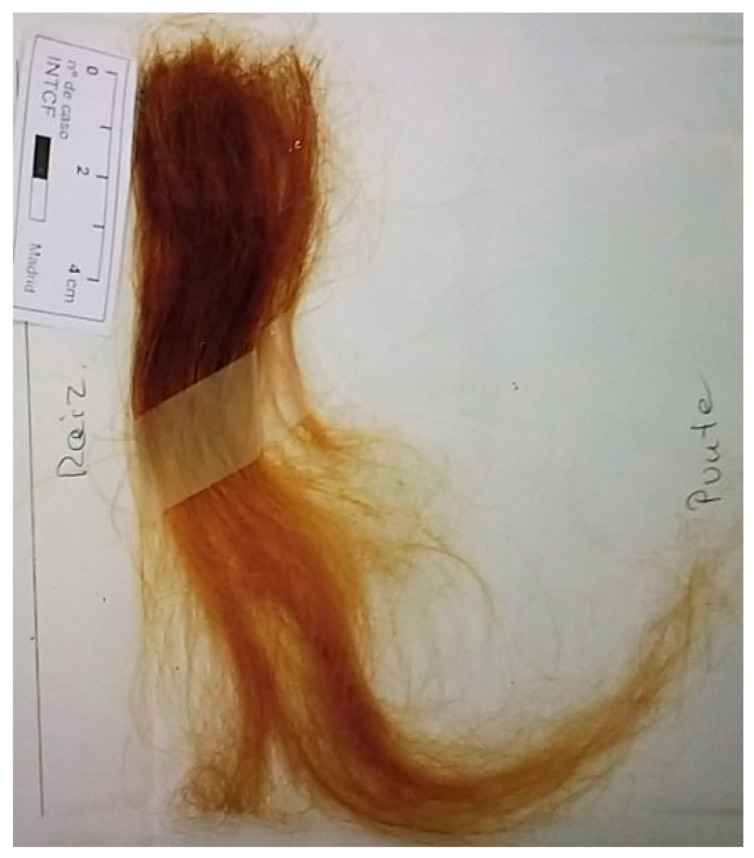
Sample of hair analyzed. An aliquot of 6 cm of length was collected, prepared for toxicological analysis and analyzed afterwards.

A female drug dealer whose age was unknown, most likely young, was charged with a crime against public health in Spain. The judicial requirement was to establish and corroborate whether the seized drug was for own consumption or not. The medical examiner of the Ministry of Justice referred one lock of brown scalp hair (30 cm each) that was dyed, from the defendant (Figure 1). An aliquot of 6 cm of length from the root was collected and prepared for toxicological analysis and was analyzed. In Spain, this type of analysis is frequently asked for reduction of penalty alleging consumption, since this fact is contemplated as an attenuator.

## 3. Materials and Methods

### 3.1. Materials

#### 3.1.1. Standards and Reagents

All reagents used were analytical grade or higher. Mobile phase solvents and additives were LC-MS grade. The Certified Reference Material of 2C-B was acquired from LGC Promochem Cerilliant (Teddington Middlesex, UK) providing either pure solutions in methanol at 1.0 mg/mL (liquid) to final concentration of working standard solutions of 2C-B (1, 0.1 and 0.01 mg/L) were prepared in methanol.

#### 3.1.2. Hair Samples

Two locks of hair samples of 6 cm long were used: (i) one lock of 20 mg blank hair was used to prepare standard solutions, collected from drug-free volunteers; and (ii) one lock of 20 mg from the hair of the real case (6 cm), which was kept in custody by the Madrid Department of the INTCF. The latter had been previously analyzed by the routine GC-MS analysis.

### 3.2. Methods

The hair samples were taken following the guidelines addressed by the Society of Hair Testing [18]. A preanalytical method developed and used in the Madrid Department of the INTCF was employed to prepare the samples for analysis by high-resolution mass spectrometry technique for the new psychoactive substances identification [10], which was UHPLC-HRMS/MS. The methodology used was previously developed for the screening of multitargeted substances [19,20], reporting their presence or absence in hair. For this work, the development of highly sensitive HRMS/MS approaches for the identification of 2C-B, constraining the fragmentation of 2C-B with the PRM mode (Parallel Reaction Mornitoring) in hair matrices was performed. The UPLC-HRMS/MS method is described in further detail in Section 3.2.2.

#### 3.2.1. Hair Sample Preparation

All the hair samples were subjected to a pretreatment stage before applying the analytical method to determine the drugs present. Different steps were used according to Matey et al. [10]. Briefly, they consisted in:

1. Double washing with 5 mL of dichloromethane.
2. The amount of hair dried (20 mg).
3. Trituration of the hair using a Precellys Tissue Homogenizer (Bertin Instruments, Montigny-le-Bretonneux, Yvelines, France). After that, the hair samples were spiked with all the internal standards.
4. Incubation of the triturated hair in 2 mL of methanol for t = 18 h, after sonication for t = 30 min.
5. Ultracentrifugation at 14,000 rpm, the supernatant was decanted and gently evaporated to dryness prior to its reconstitution in 100 µL methanol in a microvial.
6. Injection of 1 µL of the extract into the column head of the liquid chromatograph-mass spectrometer.

#### 3.2.2. Instrumental Methods

As an initial method of analysis by UHPLC-HRMS/MS methodology, a single 1 µL aliquot was injected into a Vanquish Flex Binary UHPLC system, coupled with an Orbitrap Q Exactive Focus system (provided by Thermo Fisher, Hemel Hempsted, Hertfordshire, UK). The parameters for setting the instrumentation are the same reported in Matey et al. [10]. Briefly, the stationary phase consisted in a phenylhexyl column (100 mm × 2.1 mm × 2.6 µm) and the mobile phase consisted in one initial eluent A, which was made of ammonium format 2 mM with 0.1% formic acid at pH 3. The elution was isocratic at the beginning, followed by a gradient elution, adding increasingly the eluent B, performed at a flow rate set to 0.5 mL/min. This eluent B consisted in a mixture of acetonitrile/methanol (50:50, *v/v*) and 1% of the eluent A. The gradient was programmed as follows: 0–1.0 min 1% eluent B, 1.0–10.0 min to 99% eluent B, 10–11.5 min to 1% eluent B and 11.5–13.5 min hold 1% eluent B. An electrospray ionization (H-ESI II) set in the positive ionization mode was performed. The settings for full scan ion monitoring were the same reported in the studies performed by Hans H. Maurer et al. [19,20].

Two different approaches were explored for the identification of 2C-B in hair:

(i) An initial method of target screening (TS), consisting in a multitarget analysis coupled with the mass spectrometer set for full-scan ion monitoring (FS), with data-dependent acquisition (DDA) for fragmentation in MS/MS spectrometry. The settings for TS were positive/negative switching ionization mode with FS and a subsequent DDA mode, modifying the inclusion list for different NPS and their metabolites. A screening method was applied, which was validated in terms of selectivity (given by the hyphenation of a high resolution separation technique with an exact mass spectrometer), limits of detection (LOD) and limits of identification (LOI). In this UHPLC-HRMS/MS method approach, all the resulting chromatograms and spectra were compared to the ones available at the INTCF library, using the software Trace Finder (TF) Forensic Research 4.1 (Thermo Fisher Scientific Inc., Waltham, MA, USA). It contains nowadays more than 1550 standards stored up in the database, with a particular section of mass spectrometry (compound database); composed of precursor ion, isotopic pattern for MS-1, plus library spectrum (collection of MS/MS spectras), and five principal fragments ions (FI) for a MS/MS acquisition, for each substance, normally the most intense ones. The 2C-B drug is also included in this database. The LOD and LOI were experimentally determined by this UHPLC-HRMS/MS approach; those values were compared with the same compound database of targeted screening method, available at the INTCF in this approach. The LOD and LOI were experimentally determined by this UHPLC-HRMS/MS approach and by comparing the fragments obtained for 2C-B.

(ii) In the second approach, one specific range for precursor ions for 2C-B was selected: full mass (FS) 253–268 *m*/*z*, with the same conditions of data-dependent acquisition for the identification in the real forensic case, additionally the mode Parallel Reaction Monitoring (PRM), with an isolation window 2 *m*/*z*, was used to identify and confirm 2C-B. 

The scan rate was 7 Hz at a resolution of 35,000 FWHM (Full With Half Maximum) and the same event of DDA settings were used in both detection approaches.

The mass spectrometer was calibrated at least every week in the positive mode, with an MRFA solution (L-methionyl-arginyl-phenylalanyl-alanine acetate) at 1 mg/L, caffeine at 2 mg/L, and Ultramark^®^ 1621 0.001% (a commercially available mixture of fluorinated phosphazenes). In the negative mode, with a solution of 2.9 mg/L sodium dodecyl sulfate, 5.4 mg/L sodium taurocholate, and Ultramark^®^ 1621 0.001% was used. Both modes had a mass range of 50 to 2000 *m*/*z*.

#### 3.2.3. Data Treatment: LOD and LOI

The LODs and LOIs values were determined using decreasing concentrations of drug-fortified blank hair samples with 2C-B as NPS at the following concentrations: 50, 25, 20, 15, 10, 5, 2, and 1 pg/mg and the above referred TF TraceFinder 4.1 and Free Style 1.3. software (Thermo Fisher Scientific Inc., Waltham, MA, USA). The LOD was defined as the lowest concentration exhibiting a MS signal, which was produced by the accurate precursor ion [20] and a chromatographic signal at least three-times the background height in the chromatogram [21,22,23,24]. The LOI was defined by two different criteria: (i) the accurate precursor ion must be detectable (LOD) [20,25] and (ii) the underlying HR-MS/MS spectrum must contain at least the two principal fragments of the reference library spectrum [26] corresponding with the lowest concentration of the analyte in the hair matrix.

## 4. Results and Discussion

UHPLC-HRMS/MS is a type of high-resolution mass spectrometry technique that was recently acquired by the Madrid Department of the INTCF. The potential of this technique is assessed in this case report by reanalyzing a previous forensic case, pursuing:

- to evaluate a simplification of the routine methodology for hair sample analysis in the INTCF laboratory. For this, a UHPLC-HRMS/MS method has been developed and applied to the same real case involving polyconsumption, which was previously analyzed by GC-MS; and, then,
- to identify 2C-B in the hair sample of the studied case, where the previous detection of ketamine suggested the potential presence of other NPS.

**Figure 2:**
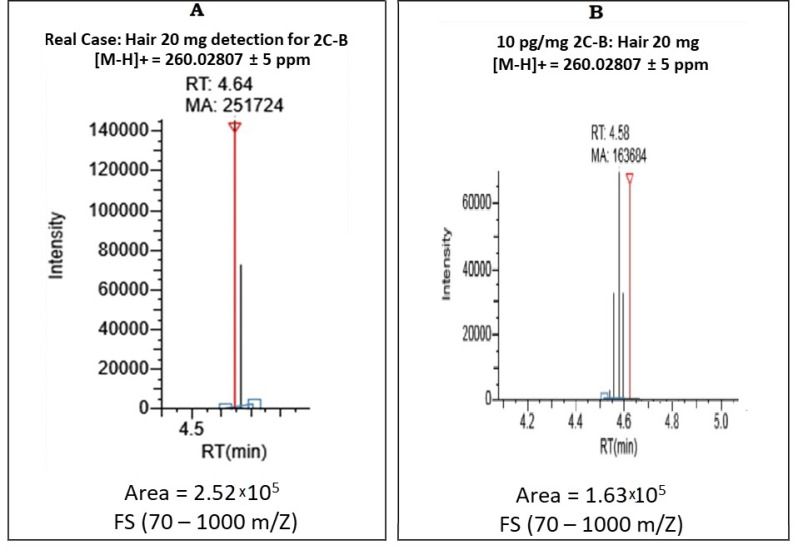
Detection of precursor ion of 2C-B analysis of accuracy mass < 5 ppm, using the UHPLC-HRMS/MS technique, in mode of acquisition Target Screening, in a real hair forensic case sample (**A**) and with a certified reference material in hair (**B**) with a concentration of 10 pg/mg in the hair matrix (LOD).

Interestingly, the analysis of this case by the Target Screening method (FS-DDA) detected the precursor ion of 2C-B (see Figure 2, chromatogram A), and the particular isotopic pattern of 2C-B (C_10_H_14_BrNO_2_) due to the different isotopes of the bromine atom. Two precursors were detected with similar intensities: C_10_H_14_[^79^Br]NO_2_ and C_10_H_14_[^81^Br]NO_2._ However, the low intensity of the aforementioned precursor ion do not exceeded the threshold limit for the fragmentation event with MS/MS with DDA; so this method was not enough for a complete identification in the real case. Thus, this first approach based on a DDA mode was complemented to complete the 2C-B identification by in a second approach by (SIM+DDA) adding a specific precursor fragmentation by PRM. The LOD and LOI were calculated for this detection approach in hair (see Section 3.2.3) and the main results obtained are compiled in Table 2.

In a second detection approach, the laboratory searched fragments of this precursor of 2C-B, with a maximum abundance. Thus, the range of selected precursors for 2C-B in the Full Scan was limited, through filtering of a quadrupole range of *m*/*z* narrower (260 ± 7.5 uma) only by the Full Scan precursor. Then, the quadrupole filter was used in a Full Scan method, which was lower than 50 uma. This method is usually called Single Ion Monitoring (SIM) because it is used specifically for a family of compounds. This ion was finally broken down to fragments ions to obtain the MS/MS spectra in different consecutive events, thanks to the PRM acquisition mode. 

**Figure 3:**
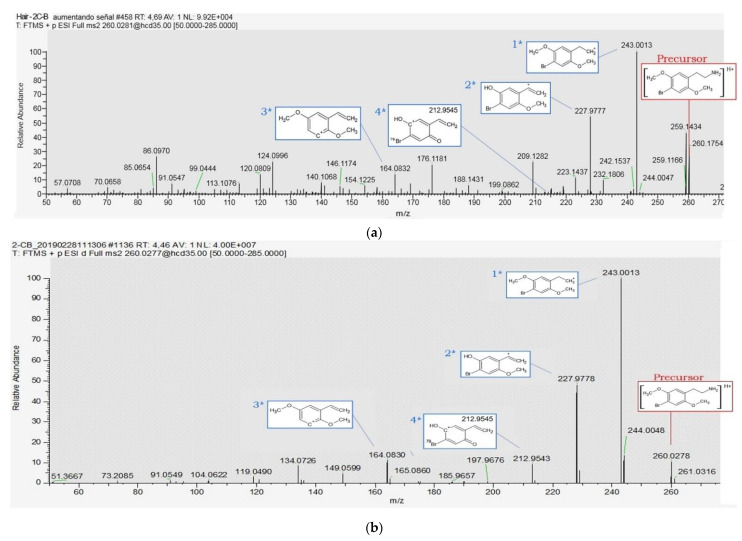
Identification of 2C-B by UHPLC-HRMS/MS by confirmation in the second approach in a real hair forensic case sample (**a**); and compared using the detection in a Certified Reference Material of 2C-B as the pure solution in methanol at 1.0 mg/L (**b**).

For the identification of 2C-B fragments, as it can be seen in the MS/MS spectra of Figure 3, the main fragments of this substance were monitored and marked in blue. In the MS/MS spectra of the real hair case, other fragments due to the matrix were also detected (see Figure 3). However, all fragments identified in the real hair case (spectra Figure 3a) were also present in the spectra of the certified reference material of 2C-B (spectra Figure 3b). In both spectra, the 2C-B fragments were detected with the same order of abundance and with an accuracy mass < 1 ppm of error. Among these fragments, the more abundant ion (1 *) was [C_10_H_12_O_2_Br]^+^ = 243.00130, attributed to a loss of a –NH_3_ group (17.0265). This has been already reported for non-targeted analysis of dimethoxy-phenylethylamines or series of 2C-X substances by Pasin Daniel et al. [14]. The other fragments (2 *, 3 * and 4 * in the Figure 3) were detected with an exact mass monitored at 227.9777, 212.9545, and 164.0830. These MS/MS fragments, their chemical structures, chemical formula, theoretical, and measured exact masses and their errors are compiled in Table 3.

**Figure 4:**
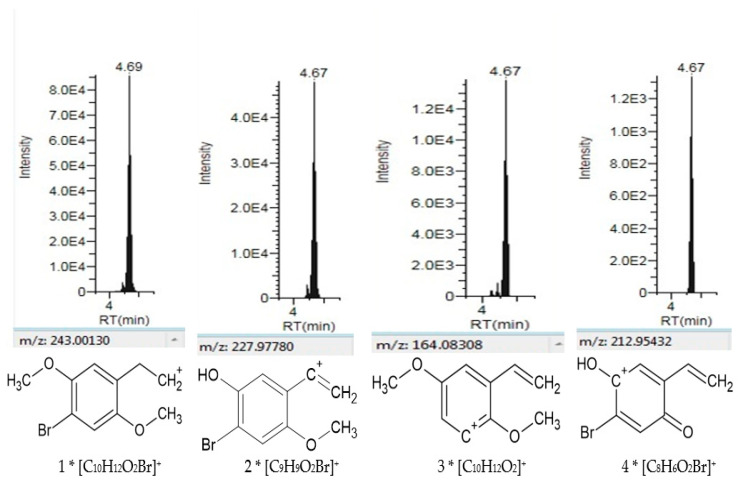
Identification of the main fragments of 2C-B by UHPLC-HRMS/MS by confirmation using a second detection approach in the real hair forensic sample using the PRM mode.

The fragments obtained in the PRM mode in the real case were detected at the same retention time (merged) for the four main fragments of 2C-B, as shown in Figure 4.

The results obtained demonstrated the positive detection of 2C-B, its isotopic profile, and the MS/MS fragments ions identification. Finally, despite this being a qualitative method where only selectivity and LOD/LOI were assessed, the estimation of the concentration of 2C-B was estimated by considering the LOI of the FS + DDA detection approach, which was 50 pg/mg (see Figure S1), whereas the LOD was 10 pg/mg. Therefore, the concentration in the real sample was estimated to be in the range 10–50 pg/mg. Nowadays, a quantitative method is being developed at the INTCF in order to validate and reliably quantify this NPS using the most sensitive and selective detection approach. 

## 5. Conclusions

In this particular case, where the identification of the psychedelic 2C-B in hair samples was pursued, only a presumptive detection was carried out because a single precursor had to be confirmed by HRMS/MS. With these types of cases, efficient ways for the detection of ultratrace concentrations were used, such as the detection approach based on parallel monitoring reaction (PRM). Then, the other two different detection approaches were explored for this NPS identification by HRMS/MS. Considering the LOI values using the Target Screening approach (FS+DDA), the concentration estimated was as low as 10–50 pg/mg. This concentration range corresponds to the average of repeated consumption in the 6–7 months prior to cutting the analyzed hair length. Up to now, there is no information available in the reviewed literature showing a positive identification of 2C-B in real hair samples. Therefore, the estimated concentration (10–50 pg/mg) is relevant to develop and validate future quantitative methods based on HRMS/MS. 

Complementarily, in this case, the identification of 2C-B was related to a polyconsumption case. This relationship can also be observed in previous studies where the detection of one drug (e.g., ketamine) leads to the consideration that other substances may have been consumed concomitantly [10,18]. However, further studies need to be carried out to assume that 2C-B is related to polyconsumption.

## Figures and Tables

**Table 1:** Chemical classification of 2C-B and its analogues as dimethoxy-phenylethylamines.

Chemical Core	Name and Molecular Formula	R	R_1_	R_2_	R_3_
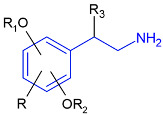 Dimethoxy-phenylethylamines	2C-B (C_10_H_14_BrNO_2_)	Br	CH_3_	CH_3_	H
	2C-D (C_11_H_17_NO_2_)	CH_3_	CH_3_	CH_3_	H
	2C-E (C_12_H_19_NO_2_)	CH_2_-CH_3_	CH_3_	CH_3_	H
	2C-H (C_10_H_15_NO_2_)	H	CH_3_	CH_3_	H
	2C-I (C_10_H_14_INO_2_)	I	CH_3_	CH_3_	H
	2C-N (C_10_H_14_N_2_O_4_)	NO_2_	CH_3_	CH_3_	H
	2C-P (C_13_H_21_NO_2_)	CH_2_-CH_2_-CH_3_	CH_3_	CH_3_	H
	C-T-2 (C_12_H_19_SNO_2_)	S-CH_2_-CH_3_	CH_3_	CH_3_	H
	2C-T-7 (C_13_H_21_SNO_2_)	S-CH_2_-CH_2_-CH_3_	CH_3_	CH_3_	H
	2C-T-2 (C_12_H_18_SNO_2_F)	S- CH_2_-CH_2_-F	CH_3_	CH_3_	H
	bk 2C-B (C_10_H_12_BrNO_3_)	Br	CH_3_	CH_3_	O
	bk 2C-I (C_10_H_12_INO_3_)	I	CH_3_	CH_3_	O
	Methoxy- 2C-B (C_11_H_15_BrNO_3_)	Br	CH_3_	CH_3_	O-CH_3_
	2C-B-Fly (C_12_H_14_BrNO_2_)	Br	Furan	Furan	H
	2C-E-Fly (C_14_H_19_NO_2_)	CH_2_-CH_3_	Furan	Furan	H
	2C-EF-Fly (C_14_H_18_NO_2_F)	CH_2_-CH_2_-F	Furan	Furan	H
	2C-I-Fly (C_12_H_14_INO_2_)	I	Furan	Furan	H
	2C-T-7-Fly (C_15_H_21_SNO_2_)	S-CH_2_-CH_2_-CH_3_	Furan	Furan	H

**Table 2:** Limit of detection (LOD) and limit of identification (LOI) of 2C-B by UHPLC-HRMS/MS by the FS + DDA detection approach.

LOD	LOI
pg/mg	pg/mg
Precursor [M-H]	Fragments (MS/MS)
MS^1^	MS^2^
10	50

**Table 3:** Main fragments of 2C-B identified and ordered by abundance. Data were acquired using UHPLC-HRMS/MS with the second detection approach (SIM + DDA). Error mass were calculated using theoretical mass-to-charge, with results of fragments in four decimals.

MS/MS Fragments				
Number by Abundance	Formula [M-H]^+^ Products	Theoretical [M-H]^+^ Products	Measured [M-H]^+^ Products	Error Mass (ppm)
1 * 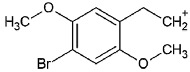	[C_10_H_12_O_2_Br]^+^	243.0015	(A) 243.0013 (B) 243.0013	−0.82
2 * 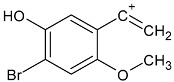	[C_9_H_9_O_2_Br]^+^	227.9778	(A) 227.9777 (B) 227.9778	−0.44 0.00
3 * 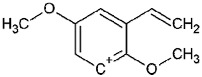	[C_10_H_12_O_2_]^+^	164.0832	(A) 164.0832 (B) 164.0832	0.00
4 * 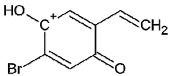	[C_8_H_6_O_2_Br]^+^	212.9545	(A) 212.9543 (B) 212.0543	−0.94

## Data Availability

The data presented in this study are available on request from the corresponding author. The data are not publicly available due to institutional and national data sharing restrictions.

## Supplementary Materials

The following are available online at https://www.mdpi.com/article/10.3390/toxics9070170/s1, Figure S1: LOI 50pg/mg by 2C-B in Target Screening method.
