# Supplementary material for: Identification of 2C-B in Hair by UHPLC-HRMS/MS. A Real Forensic Case
Source: Toxics. 2021 Jul 15;9(7):170. doi: 10.3390/toxics9070170 (PMC8309701; doi:10.3390/toxics9070170)
Supplement: Supplementary file 1 [file toxics-09-00170-s001.zip › toxics-1288492-supplementary.pdf]

toxics

MDPI

Case Report

Supplementary Materials: Identification of 2C-B in Hair by UHPLC-HRMS/MS. A Real Forensic Case

José Manuel Matey, Adrián López-Fernández, Carmen García-Ruiz, Gemma Montalvo, Félix Zapata and María A. Martínez

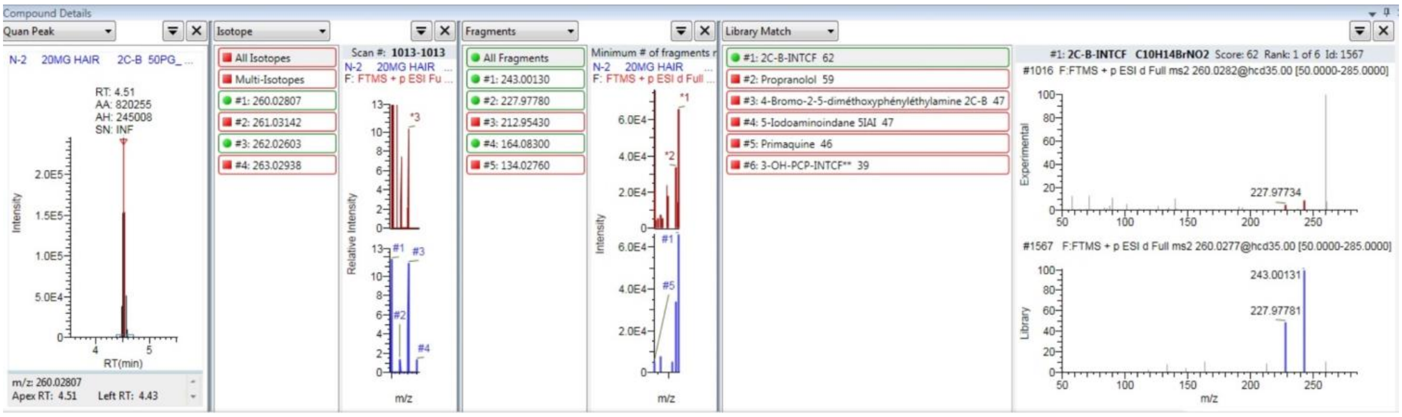

Figure S1. LOI 50pg/mg by 2C-B in Target Screening method.
